# The Kinematics of Plant Nutation Reveals a Simple Relation between Curvature and the Orientation of Differential Growth

**DOI:** 10.1371/journal.pcbi.1005238

**Published:** 2016-12-06

**Authors:** Renaud Bastien, Yasmine Meroz

**Affiliations:** 1 Department of Collective Behaviour, Max Planck Institute for Ornithology and Department of Biology, University of Konstanz, Konstanz, Germany; 2 John A. Paulson School of Engineering and Applied Sciences, Harvard University, Cambridge, Massachusetts, United States of America; INRIA, Montpellier, FRANCE

## Abstract

Nutation is an oscillatory movement that plants display during their development. Despite its ubiquity among plants movements, the relation between the observed movement and the underlying biological mechanisms remains unclear. Here we show that the kinematics of the full organ in 3D give a simple picture of plant nutation, where the orientation of the curvature along the main axis of the organ aligns with the direction of maximal differential growth. Within this framework we reexamine the validity of widely used experimental measurements of the apical tip as markers of growth dynamics. We show that though this relation is correct under certain conditions, it does not generally hold, and is not sufficient to uncover the specific role of each mechanism. As an example we re-interpret previously measured experimental observations using our model.

## Author Summary

In his writings, Darwin considered nutation, the revolving movement of the apical tip of plants, as the most widespread plant movement. In spite of its ubiquity, plant nutation has not received as much attention as other plant movements, and its underlying mechanism remains unclear. A better understanding of this presumably growth-driven process is bound to shed light on basic growth processes in plants. In the work presented here we redefine the problem by describing the kinematics in three dimensions, as opposed to the typical description restricted to the horizontal plane. Within this framework we reveal a simple picture of the underlying dynamics, where the orientation of curvature follows the orientation of maximal differential growth. This parsimonious model recovers the major classes of nutation patterns, as shown both analytically and numerically. We then discuss the limitations of classical measurements where only the movement of the apical tip is tracked, suggesting more adequate measurements.

## Introduction

During their development, plant organs display a large range of movements. These movements may be broadly divided into two classes; tropisms and nastic movements. Tropisms are the reorientation towards an external stimulus, *e.g.* light or gravity [1, 2]. Nastic movements account for endogenous, autonomous movements and are not directed towards an external stimulus. Nutation, often called circumnutation, is a particular class of nastic movements, in which the plant organ successively bends in different directions, resulting in an apparent oscillatory swinging motion. Despite its ubiquity among plant movements nutation has not been studied as extensively as tropisms, and the mechanism responsible for this movement, as well as its regulation, remain unclear (see [3–5] for a review).

Current theories or concepts of nutational mechanisms generally fall into two categories [6]. The first suggests the influence of external drivers such as gravity or light, where the movement stems from an overshoot during the straightening of the plant in response to the direction of gravity (or light). The second assumes an endogenous driver such as an oscillator, suggested by Darwin [7], possibly related to the growth process itself [3, 8]. Studies have shown that though gravitropism may influence and modify the observed movements, the two processes exist independently [3, 9, 10], consistent with symmetry arguments which indicate that gravitropism alone cannot induce movement outside of the plane defined by the main axis of the plant and the direction of gravity [11, 12]. Together with the fact that nutation is observed in the absence of light, this suggests that external cues cannot drive nutation. Interestingly, Brown [3, 5] postulated that since nutation does not present any significant evolutionary benefit, it may be the consequence of some fundamental mechanism in the growth process. Observations of rice coleoptile mutations (*lazy*) that grow normally yet do not exhibit nutation [10], suggest that growth alone may not be sufficient to generate nutation [3, 8]. Therefore based on the present literature, the strongest hypothesis remains a growth-driven endogenous oscillator.

**Fig 1:**
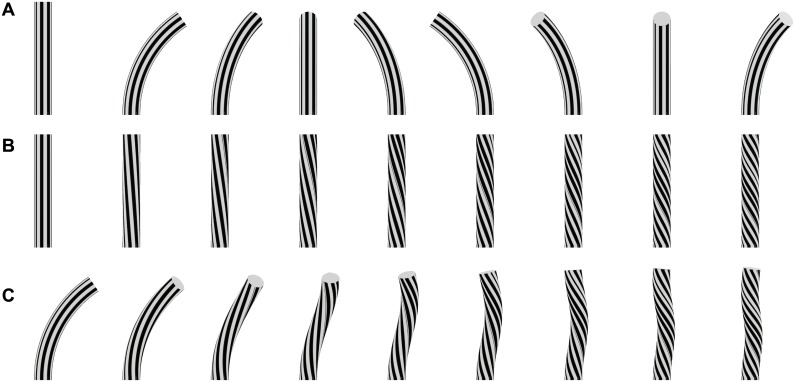
A cylinder is used as a simplified representation of an organ. Lines parallel to the median line of the organ are drawn on the surface. A. The organ can be curved in different direction of space without material torsion: parallel lines on the surface remain parallel. B. Material torsion of increasing intensity are applied on the organ (from left to right). The torsion does not change the shape of a straight cylinder. However parallel lines at the surface of the organ are no longer parallel to the median line, but are tilted. C. Material torsion of increasing intensity (from left to right) is applied to a curved organ. The organ does not lie in a plane anymore but the curvature takes different direction in the 3D space. The organ displays a helical shape.

We note that curvature of an elongated organ in three dimensional space can result from two different growth mechanisms, namely bending and torsion. Bending can result from the differential growth of the opposite sides of an organ [13, 14], i.e an initially straight organ will bend towards the direction of minimal growth. Studies have mainly focused on the case where the organ is curved in the same (vertical) plane as that of the differential growth, restricting movement to that plane only. However the plane of curvature should change when it is not in the same plane as that of the differential growth, producing movement in the horizontal (apical) plane. It is instructive to note that lines drawn on the surface of the organ, parallel to its main axis, will remain parallel to the main axis during its growth regardless of the direction of curvature, as shown in Fig 1.A. The second mechanism, torsion, is responsible for the movement of twining plants [15, 16], and is due to the helical arrangement of cells around the main axis of the plant, possibly due to the torsional arrangement of cellulose [17]. In this case parallel lines drawn on the surface of the organ will take a helical form around the organ during its growth, as shown in Fig 1, e.g. the cotyledon on top of a hypocotyl will rotate. However this process can lead to a 3D curved organ only if the organ is already initially curved, and furthermore results in a helical form (see Fig 1.C). Moreover, observations of torsion in nutating plant organs have been found to be too slow to account for the observed nutation [18, 19]. These observations hint that the dominant growth mechanism underlying nutation is differential growth under the action of an internal oscillator [19]. This internal oscillator could then be related to the auxin dynamics or the sensitivity of the membrane to auxin. Indeed a relation has been found between oscillations in ion fluxes and nutation [20, 21]. Moreover there are some reports of relationships between nutation and biological rhythms [22, 23], demonstrating genetically that the circadian clock controls nutation speed [24]. Together, these results suggest that genetically regulated rhythmical membrane transport processes are central to plant nutation, and may play the role of an internal oscillator [5].

In this study we consider nutation as a growth-driven process, in line with previous work on tropisms and differential growth [11–14] where mechanical effects such as buckling and instabilities are disregarded. We then focus on the relation between internal oscillatory growth patterns and the observed movement.

Attempts have already been made to develop a mathematical model of nutation, but the full three-dimensional geometry of the organ has been neglected, resulting in an incomplete kinematic description [25]. The existing models account only for the kinematics of the apical tip, and this has been shown to be insufficient to understand the underlying mechanisms, since geometrical and local effects are neglected [1, 13].

A similar problem exists in the experimental measurement of nutation, where the full dynamics of plants in three-dimensional space and time are rarely taken into account. It is common, instead, to track the projection of the apical part of the organ in the plane orthogonal to the gravitational field (defined here as the horizontal plane *P*_*a*_) [18, 26]. Such measurements carried out on different species and organs [7, 26] exhibit disorganized patterns (zig-zag shaped), and organized patterns (for instance, elliptical patterns, and the limits of these patterns, *e.g.* a circle or a line) [8, 18, 22, 27, 28]. The interpretation of these measurements remains unclear, since the relation between the differential growth pattern and the kinematics in space and time is not clearly defined.

Here we will state a growth-driven parsimonious model couched within a three-dimensional geometrical framework, accounting for observed classes of movement patterns. The analysis is done both analytically and through numerical simulations. The model’s limitations are also discussed. Lastly, the model is applied to existing experimental observations, and the relevance of apical (horizontal) measurements is discussed. The details of all calculations are given in the appendix. In addition, an interactive simulator is available online [29]. Predefined solutions are accessible through the numerical key of the keyboard and are referenced throughout the manuscript.

## Models

### Geometrical Description

The geometric framework used to describe the kinematics of tropisms [11, 12, 14] is only sufficient to describe growing elongated organs in a single plane and is therefore inadequate here. Unlike the movements observed for example in gravitropism where the curve is constrained to a unique plane, in nutation the organ is curved along different planes in 3D space (Fig 1.A).

**Fig 2:**
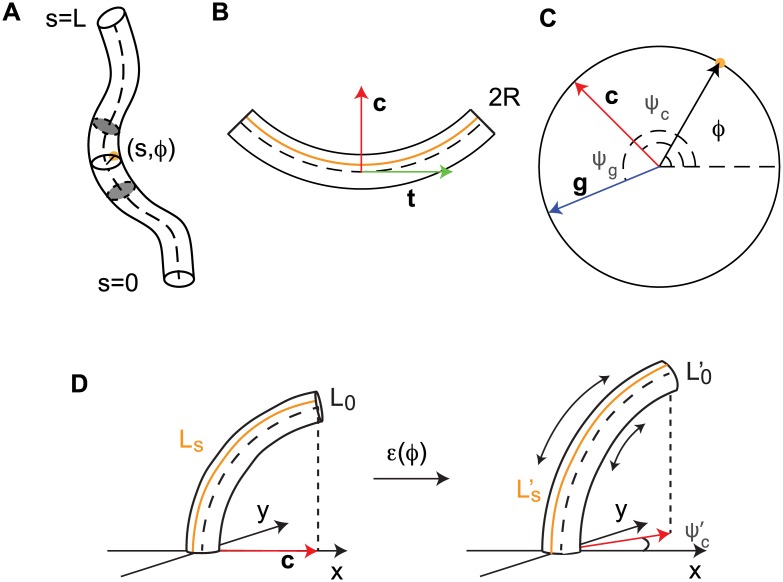
**A. A plant organ in 3D, described in cylindrical coordinates**. The parameter *s* runs along the abscissa of the organ, with the base at *s* = 0 and apex at *s* = *L*. A cross-section of of the organ is shown, with a point, in orange, on its circumference defined by the pair (*s*, *ϕ*). Here *s* states where the cross-section is along the organ, and *ϕ* determines the orientation from an arbitrarily chosen starting point. B. An element of the organ shown in A, delimited by two grey cross-sections, projected onto the plane defined by the vectors **t** (the tangent, in green), and **c** (the normal in the principal direction of curvature, in red). In orange, a segment parallel to the median line is defined by *ϕ* C. The cross section of the organ shown in A in the plane perpendicular to **t**. Again, an element on the circumference of the organ is defined by the angle *ϕ*. The direction of the vector normal in the principal direction of curvature, **c** in red, is defined by the angle *ϕ* = *ψ*_*c*_. The principal direction of the differential growth, defined by the vector **g** in blue, is defined by the angle *ϕ* = *ψ*_*g*_.Due to the cylindrical symmetry considered for the organ, the direction given by *ϕ* = 0 is defined arbitrarily but continuously on the whole organ. When the organ is not curved, the direction given by any *ϕ* defines a straight line along the organ parallel to the main axis of the plant. D. An element cylinder curved in the 3D space, here in the direction *ψ*_*c*_ = 0. Its median length is given by *L*_0_, while a segment on the surface has a length *L*_*s*_(*ϕ*). After a strain *ϵ*(*ϕ*) is applied so that the length of each segment on the surface is now $L_{s}^{\prime }\left(\phi \right)$. The cylinder is now curved in a different direction, $\psi_{c}^{\prime }$, while the curvature is also modified, *C*′. Finally the length of the median line is given by $L_{0}^{\prime }$. See S1 Video.

**Fig 3:**
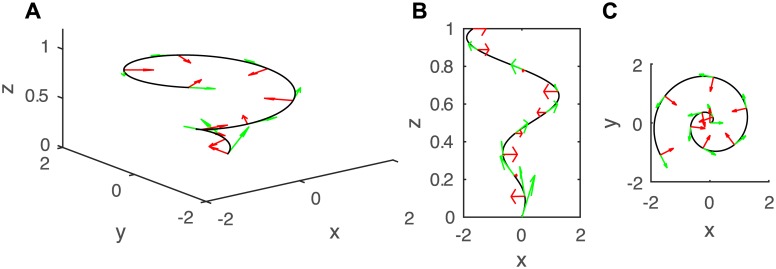
**A. A curve in 3D space** (*x*, *y*, *z*). Here, a generalized spiral has been chosen as it provides a nice and simple illustration of an organ curved in different planes in 3D. The curve is described at each point by two vectors, as defined in Fig 2: the tangent and normal to the curve in the plane of the principal direction of curvature, **t** and **c** (shown in green and red respectively). The vectors are orthogonal to each others. B. and C. present the projections of the curve onto the (*x*, *z*)and (*y*, *z*) planes respectively. See S1 Video.

We start by introducing a few assumptions and definitions which will be essential for the construction of our three dimensional model (Fig 1). The organ is assumed to be cylindrical with a constant radius *R* along the organ. It is assumed that no shear growth is observed, so the cross section remains in plane. The organ is described by the curvilinear abscissa *s* along the median line. Each point at the surface of the organ is then defined via cylindrical coordinates (*s*, *ϕ*) where *s* is its position along the abscissa, and *ϕ* is the angle of the point on the cross section, relative to an arbitrarily chosen direction. This description is depicted in Fig 2.A and in S1 Video. In order to fully describe the curvature of an organ curved in an arbitrary direction in space, it is first necessary to define two vectors: **t**, the tangent to the median line, and **c**, the normal (perpendicular) to the median line, as shown in Fig 2.B. The orientation of the latter in the cross section, *ψ*_*c*_(*t*), is in the same plane as the principal direction of curvature (see Fig 3). This means that for each element of the curve, the curvature is maximal in the plane defined by the vectors **t** and **c** (see Fig 2.B). Fig 2.C shows a cross section of the shoot, by definition in the plane orthogonal to **t**, defining the orientation *ψ*_*c*_ of the principal direction of curvature **c**.

### Model

#### Slaving of the principal direction of curvature to direction of maximal growth

Given the geometrical framework presented here, we now bring a parsimonious model describing nutation kinematics based on basic mechanic and geometric arguments. We recall that for simplicity we assume an organ with constant radius *R*. We consider the elongation strain rate $\dot{\epsilon }\left(\phi ,s,t\right)$ defined at each point (*ϕ*, *s*) along the surface of the organ, describing a compatible transformation of a cylindrical organ of radius R and curvature *C*(*s*, *t*) in the direction *ψ*_*c*_(*s*, *t*) into a cylinder of radius *R* and curvature *C*′(*s*, *t*) in the direction $\psi_{c}^{\prime }\left(s,t\right)$. We now proceed to represent the strain rate $\dot{\epsilon }\left(\phi ,s,t\right)$ as a function of the the variation of curvature $\frac{dC(s,t)}{dt}$ and the variation of the principal direction of curvature $\frac{d\psi_{c}\left(s,t\right)}{dt}$, in turn leading to equations of motion.

We start with an infinitesimal cylindrical element where the curvature *C*(*s*, *t*) = *C* and its principal direction *ψ*_*c*_(*s*, *t*) = *ψ*_*c*_ are assumed to be constant along the median line of length *L*_0_ (Fig 2.D). Since we are discussing an infinitesimal element, we drop the dependence on *s*, the position along the median line of the whole organ. We also drop the temporal dependence *t* for simplicity. The length of a segment running along the surface of the infinitesimal curved cylinder, parallel to the median line, depends on its angle *ϕ* relative to the principal direction of curvature *ψ*_*c*_:

$$\begin{array}{c} L_{s}\left(\phi \right)=L_{0}\left(1-CR\cos\left(\phi -\psi_{c}\right)\right). \end{array}$$ (1)

We note that for a line in the principal direction of curvature, i.e. at the inner part of the curve, the length is minimal, *L*_*s*_(*ϕ* = *ψ*_*c*_) = *L*_0_(1 − *CR*), while it is maximal for a line in the outer part of the curve *L*_*s*_(*ϕ* = *ψ*_*c*_ + *π*) = *L*_0_(1 + *CR*). A similar relation holds for the cylinder after deformation, namely $L_{s}^{\prime }\left(\phi \right)=L_{0}^{\prime }(1-C^{\prime }R\cos(\phi -\psi_{c}^{\prime }))$.

We note that the elongation strain *ϵ*(*ϕ*) at a specific point along the cylinder is defined as the ratio between the elongation length $L_{s}^{\prime }\left(\phi \right)-L_{s}\left(\phi \right)$ and the original length *L*_*s*_(*ϕ*)

$$\epsilon \left(\phi \right)=L_{s}^{\prime }\left(\phi \right)/L_{s}\left(\phi \right)-1$$ (2)

Similarly the average elongation strain rate defined as

$$E=\frac{1}{2\pi }\int_{-\pi }^{\pi }d\phi \epsilon (\phi )$$ (3)

is related to the ratio of the median lengths. Substituting $L_{0}^{\prime }=L_{0}\left(E+1\right)$, the relation for the cylinder after deformation reads:

$$\begin{array}{c} L_{s}^{\prime }\left(\phi \right)=\left(1+E\right)L_{0}\left(1-C^{\prime }R\cos\left(\phi -\psi_{c}^{\prime }\right)\right). \end{array}$$ (4)

Substituting eqs 1 and 4 into Eq 2 yields an expression for the elongation strain *ϵ*(*ϕ*):

$$\begin{array}{c} \epsilon \left(\phi \right)=\left(1+E\right)\frac{1-C^{\prime }R\cos\left(\phi -\psi_{c}^{\prime }\right)}{1-CR\cos(\phi -\psi_{c})}-1. \end{array}$$ (5)

We now introduce time by considering an infinitesimal time step *dt* and substituting the first order differentials $\epsilon \left(\phi \right)=\dot{\epsilon }\left(\phi \right)dt$, $E\left(\phi \right)=\dot{E}\left(\phi \right)dt$, *C*′ = *C* + *dC*, and $\psi_{c}^{\prime }=\psi_{c}+d\psi_{c}$, assuming that terms with second order infinitesimals are negligible, and noting that cos(*ϕ* − (*ψ*_*c*_ + *dψ*_*c*_)) = cos(*ϕ* − *ψ*_*c*_) + sin(*ϕ* − *ψ*_*c*_)*dψ*_*c*_. We use the common dot notation for strain rates. Rearranging and reintroducing the explicit dependence on *t* into the notation, Eq 5 now reads:

$$\dot{\epsilon }\left(\phi ,t\right)=\dot{E}\left(t\right)+\frac{\sin\left(\phi -\psi_{c}\left(t\right)\right)\frac{d\psi_{c}\left(t\right)}{dt}C\left(t\right)R+R\cos\left(\phi -\psi_{c}\left(t\right)\right)\frac{dC(t)R}{dt}}{1-C\left(t\right)R\cos\left(\phi -\psi_{c}\left(t\right)\right)}.$$ (6)

This equation relates the elongation rate of a fiber, or a segment running along the organ surface, to the rate of change in curvature magnitude $\frac{dC(t)}{dt}$ and direction $\frac{d\psi_{c}}{dt}$.

**Fig 4:**
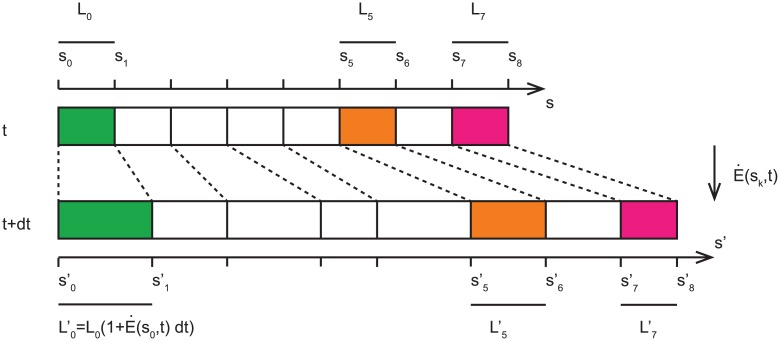
An illustration of the material derivative. As a simplification, a plant organ can be seen as a succession of elongating elements. Their position along the curvilinear abscissa is given by the lowest position of the element *s*_*i*_, and its length defined by the position of the subsequent element; *L*_*i*_ = *s*_*i*+1_ − *s*_*i*_. For example the 5th element, in orange, starts at point *s*_5_, and its length is defined by *L*_5_ = *s*_6_ − *s*_5_. For simplicity we assume that at time *t*, all elements have identical length of *L*_*i*_ = *L*_0_, and an elongation rate $\dot{E}\left(s,t\right)$ is applied to each element of the organ during a time *dt*. The elongation of each element is then $L_{i}^{\prime }=L_{i}(1+\dot{E}\left(s,t\right)dt)$. The difference in length for each element is then $L_{i}^{\prime }-L_{i}=L_{i}\dot{E}\left(s,t\right)dt$. In the example of the first element (in green), the position is not modified, i.e. $s_{0}^{\prime }=s_{0}$, since this is the basal point which is fixed. However the increase in the element’s length shifts the positions of all other elements, e.g. the next element originally at position *s*_1_ is now at a position $s_{1}^{\prime }=s_{1}+L_{0}\dot{E}\left(s_{0},t\right)$. The second element is pushed by the elongation of both the basal element at *s*_0_ and the next element at position *s*_1_. Following this argument, the displacement of any element is then given by the sum of the elongations of all previous elements, $s_{n}^{\prime }-s_{n}=\sum_{k=0}^{n}L_{0}\dot{E}\left(s_{k},t\right)dt$. The velocity of an element is then given by the displacement of this element $s_{n}^{\prime }-s_{n}$ divided by the time interval dt. In the continuous limit, *L*_0_ → *ds*, this velocity is given by $v\left(s,t\right)=\int_{0}^{s}ds^{\prime }\dot{E}\left(s^{\prime },t\right)$. It is important to note that even if locally no elongation occurs, for example for the last element shown in pink with $\dot{E}\left(s_{n},t\right)=0$, the element is still displaced due to the displacement of previous elements. The change of curvature along the organ can be understood in a similar fashion (see Figure 1 in [14]).

We recall that this relation does not exhibit an explicit dependence on *s* since the argument was for an infinitesimal cylindrical element. The full organ is then made up of consecutive deforming (elongating) infinitesimal elements, each with dynamics described by Eq 6. We note that one cannot just yet reintroduce the dependence on *s*, the position along the median of the organ relative to the base, since as the organ deforms and elongates the positions of the constituting elements move along the organ. The variable *s* is relative to the base only, and not to the material elements. Therefore when considering the whole organ we introduce the material derivative, co-moving with each element of the organ [1, 13, 14] (see Fig 4 for a description of the material derivative):

$$\frac{D}{Dt}=\frac{\partial }{\partial t}+v\left(s,t\right)\frac{\partial }{\partial s}$$ (7)

where *v*(*s*, *t*) is the velocity of the average growth-induced displacement of each element at *s* at time *t*, and is defined as the integral of the average elongation rate on the median line $\dot{E}\left(s,t\right)$ defined in Eq 3:

$$v\left(s,t\right)=\int_{0}^{s}ds^{\prime }\dot{E}\left(s^{\prime },t\right).$$ (8)

Eq 6 can be modified to account for a cylindrical organ of constant radius, that is elongating and where the curvature at each point along the median line of the organ *s* is modified in intensity and direction. We now rewrite Eq 6 to account for the elongation strain rate along the whole organ by replacing time derivatives with the material derivative in Eq 7, and reintroducing the explicit dependence on *s*:

$$\dot{\epsilon }\left(\phi ,s,t\right)=\dot{E}\left(s,t\right)+\frac{\sin\left(\phi -\psi_{c}\left(s,t\right)\right)\frac{D\psi_{c}\left(s,t\right)}{Dt}C\left(s,t\right)R+\cos\left(\phi -\psi_{c}\left(s,t\right)\right)\frac{DC(s,t)R}{Dt}}{1-C\left(s,t\right)R\cos\left(\phi -\psi_{c}\left(s,t\right)\right)}.$$ (9)

Let us note that Eq 9 has two main contributions that dominate in orthogonal planes. In the plane parallel to the principal direction of curvature, i.e *ϕ* = *ψ*_*c*_ or *ϕ* = *ψ*_*c*_ + *π*, we have sin(*ϕ* − *ψ*_*c*_(*s*, *t*)) = 0 and cos(*ϕ* − *ψ*_*c*_(*s*, *t*)) = ±1, therefore the elongation strain rate is only affected by the change in curvature $\frac{DC(s,t)}{Dt}$. In the orthogonal plane, i.e *ϕ* = *ψ*_*c*_ ± *π*/2, the elongation strain rate is only affected by the change in the orientation of the curvature plane $\frac{D\psi_{c}}{Dt}$. It is instructive to consider this in terms of differential growth, generally defined as the difference between elongation strain rates at opposite sides of the organ, divided by the average strain rate:

$$\Delta \left(\phi ,s,t\right)\equiv \frac{\dot{\epsilon }\left(\phi ,s,t\right)-\dot{\epsilon }\left(\phi +\pi ,s,t\right)}{2\dot{E}\left(s,t\right)}$$ (10)

Note that Δ(*ϕ*, *s*, *t*) = 0 implies that growth is the same on either side in this plane, while Δ(*ϕ*, *s*, *t*) = ±1 means that all of the growth occurs on one side of the organ or the other. We can now define the differential growth in the plane parallel to the principal direction of curvature *ψ*_*c*_:

$$\Delta_{\|}\left(s,t\right)\equiv \Delta \left(\psi_{c},s,t\right)=\frac{\dot{\epsilon }\left(\psi_{c}\left(s,t\right),s,t\right)-\dot{\epsilon }\left(\psi_{c}\left(s,t\right)+\pi ,s,t\right)}{\dot{E}\left(s,t\right)},$$ (11)

and in the orthogonal plane:

$$\Delta_{⊥}\left(s,t\right)\equiv \Delta \left(\psi_{c}+\pi /2,s,t\right)=\frac{\dot{\epsilon }\left(\psi_{c}\left(s,t\right)+\pi /2,s,t\right)-\dot{\epsilon }\left(\psi_{c}\left(s,t\right)+3\pi /2,s,t\right)}{\dot{E}\left(s,t\right)}.$$ (12)

Substituting Eq 9 and rewriting, leads to an equivalent set of equations:

$$\frac{DC\left(s,t\right)R}{Dt}\;=\;\Delta_{\|}\left(s,t\right)\dot{E}\left(s,t\right)\left(1-C{\left(s,t\right)}^{2}R^{2}\right)∼\Delta_{\|}\left(s,t\right)\dot{E}\left(s,t\right)$$ (13)

$$\frac{D\psi_{c}\left(s,t\right)}{Dt}\;=\;\frac{1}{C\left(s,t\right)R}\Delta_{⊥}\left(s,t\right)\dot{E}\left(s,t\right)$$ (14)

Assuming the radius of curvature 1/*C*(*s*, *t*) remains large compared to the radius *R* of the organ, *i.e.* *C*(*s*, *t*)*R* ≪ 1, the quadratic prefactor in Eq 13, *C*(*s*, *t*)^2^ *R*^2^, can be neglected [14].

Eq 13 expresses the variation of curvature as a function of Δ_∥_ alone, the differential growth in the plane of the principal direction of curvature, and is identical to the equation found in the case of in-plane curvature [13, 14]. In absence of compression, $\dot{E}\left(s,t\right)\geq 0$, the curvature is controlled by Δ_∥_, and the elongation rate only modulates the intensity of the reaction.

Out of plane curvature is then fully described by the addition of Eq 14, which, similary to Eq 13, expresses the variation of the principal direction of curvature as a function of Δ_⊥_, the differential growth in the orthogonal plane. However here the intensity of the reaction is modulated by the inverse of the curvature *C*(*s*, *t*)^−1^ *R*^−1^. If the curvature of the organ is small, a small variation of differential growth in the orthogonal plane can have strong effects on the orientation of the organ. However when the organ is more curved, the differential growth in the orthogonal plane would have to push the organ more to modify its orientation.

Moreover we see that only three quantities govern the relation between growth and the resulting nutation movement: the average elongation rate on the median line $\dot{E}\left(s,t\right)$, the differential growth in the plane of curvature Δ_∥_(*s*, *t*), which expresses the curvature variation *via* Eq 13, and finally the differential growth in the orthogonal plane Δ_⊥_(*s*, *t*), which expresses the change of orientation of the plane of curvature *via* Eq 14.

It is instructive to note that, without loss of generality, it is possible to express Δ_∥_(*s*, *t*) and Δ_⊥_(*s*, *t*) as a projections of the principal direction of differential growth Δ(*ψ*_*g*_(*s*, *t*)), *i.e.* where the differential growth is maximal:

$$\Delta_{\|}\left(s,t\right)=\Delta \left(\psi_{g}\left(s,t\right)\right)\cos\left(\psi_{g}\left(s,t\right)-\psi_{c}\left(s,t\right)\right),$$ (15)

$$\Delta_{⊥}\left(s,t\right)=\Delta \left(\psi_{g}\left(s,t\right)\right)\sin\left(\psi_{g}\left(s,t\right)-\psi_{c}\left(s,t\right)\right).$$ (16)

In summary, the model presented here suggests that nutation results from the slaving of the direction of maximal curvature, *ψ*_*c*_(*s*, *t*), to the direction of maximal growth *ψ*_*g*_(*s*, *t*).

#### The effects of growth

In linear organs growth is often limited to a zone below the apex [13, 14]. Two cases are usually considered: i. when the length *L* of the organ is smaller than the length of the growth zone *L* < *L*_*gz*_, the organ elongates along its entire length, ii. when *L* > *L*_*gz*_ the growth is localized to a subapical zone.

The effects of growth on the variation of curvature, restricted to the plane of curvature, Eq 13, have been discussed in [14]. Two main destabilizing effects of the growth process have been described: i. A passive orientation drift, where the organ elongates in absence of differential growth, $\dot{E}\left(s,t\right)>0$ and Δ_∥_(*s*, *t*) = 0, so the curvature of the organ remains the same while the length of the organ is modified. The angle of the organ, relative to the vertical, passively drifts within the plane of curvature during growth. ii. A fixed curvature; when an element close to the base leaves the growth zone, $\dot{E}\left(s,t\right)=0$, the curvature cannot be modified there anymore. It has been shown experimentally that these effects are negligible due to regulation governed by proprioception [14].

Similar effects are expected to take place when the variation of the direction of curvature is considered, as in Eq 14. i. In the absence of regulation, Δ(*ϕ*) = 0, the direction of curvature cannot be modified anymore. If the organ is curved in a single plane, no modification will be observed of the shape or movement. However if an organ is curved in multiple planes, displaying a helical shape, the radius of the helix will increase. ii. As elements leave the growth zone, the direction of curvature will remain fixed. If the direction of curvature is oscillating, this will result in a helical shape of the fixed part of the plants. However those destabilizing effect does not hold the same role in the postural control of the plant, the ability of the organ to reach the vertical position and align with the direction of gravity [11, 30].

As *a priori* there is no preferred direction of curvature, only the magnitude of curvature should be regulated, and not its direction. This regulation depends mainly on proprioception.

The effects of elongation can be studied as a perturbation [14], and it is therefore proposed first to neglect the elongation of the stem on the resulting pattern, so that $\dot{E}$ and the length of the organ *L* = *L*_0_ are constant. Once the movement is clearly defined without growth, it will be simple to discuss the perturbation due to growth.

#### Measurements of the apical tip in the horizontal plane

As mentioned earlier, it is common to measure nutation by tracking the projection of the apical part of the organ in the horizontal plane [18, 26]. However the kinematics described in this paper show that it might be difficult to understand these measurements without a measure of the whole shape and kinematics of the plants, *i.e.* many shapes can result in a similar position of the apical tip. In order to relate this measurement to inner mechanism of plants, a unique relation between the shape of the organ and the position of the apical tip needs to be assumed. Constraints on the shape need to be stated explicitly so that only one 3D shape of an organ can be mapped to a single position of the apical tip in the horizontal plane. The following set of constraints is proposed:

The organ is assumed to be curved inside a single plane with constant curvature all along the organ. The variation of curvature and of the principal direction of curvature are also considered to be the same all along the length of the organ that undergoes transformation. Lastly, if the elongation is not measured, the effects due to the change of length of the organ are disregarded.

The entire organ is then considered as a whole. The associated curvature and differential growth considered are give by their respective combined effects on all parts of the organ. This set of constraints is not unique but has been chosen for the small set of constraints that need to be assumed.

#### Variation of the principal direction of the differential growth *ψ*_*g*_(*s*, *t*) as an internal oscillator

The variation of the principal direction of the differential growth *ψ*_*g*_(*s*, *t*) provides a natural way to implement an endogenous oscillator, since it is the only known growth driven process occurring parallel to the horizontal plane. To date there are no exact experimental observations of the temporal variation of *ψ*_*g*_(*t*), however the existence of a linear oscillator seems consistent with measurements made on opposite sides of an organ [19, 31], where the differential growth oscillates periodically from one side to the other.

Moreover, the model allows to extract *ψ*_*g*_(*s*, *t*) and $\Delta \left(\psi_{g}\left(t\right),t\right)\dot{E}\left(t\right)\left(s,t\right)$ from 3D experimental data of the curvature *C*(*s*, *t*) (and therefore also *R* and *ψ*_*c*_(*s*, *t*)). Substituting eqs 13 and 14 in eqs 15 and 16 leads to the following relations:

$$\psi_{g}\left(s,t\right)-\psi_{c}\left(s,t\right)=\arctan\left(\frac{C\left(s,t\right)R\frac{D\psi_{c}\left(s,t\right)}{Dt}}{\frac{DC(s,t)R}{Dt}}\right)$$ (17)

and

$$\Delta \left(\psi_{g}\left(s,t\right),s,t\right)\dot{E}\left(s,t\right)=\sqrt{{\left(\frac{DC(s,t)R}{Dt}\right)}^{2}+{\left(C\left(s,t\right)R\frac{D\psi_{c}\left(s,t\right)}{Dt}\right)}^{2}}.$$ (18)

Considering Eqs 13 and 14 we note that for an oscillatory movement such as nutation, the direction of curvature changes over time, i.e. $\frac{D\psi_{c}\left(s,t\right)}{Dt}\neq 0$. In absence of compression the median elongation rate $\dot{E}\left(s,t\right)$ is always positive, and cannot control the direction of *ψ*_*c*_(*s*, *t*) [14]. Therefore the oscillatory movement can only be governed by Δ_∥_(*s*, *t*).

We now consider two basic cases for the functional form of *ψ*_*g*_(*s*, *t*), and analyze the ensuing organ movement. The simplest case is given when the direction of the differential growth is fixed, *ψ*_*g*_(*s*, *t*) = *ψ*_*g*_. The orientation of the organ is modified so that the principal direction of curvature *ψ*_*c*_(*s*, *t*) aligns with the direction of the differential growth *ψ*_*c*_(*s*, *t*) (see Appendix section 1.1). Despite its triviality, this result sheds light on the behavior of the out-of-plane curvature driven by differential growth. The organ tries to align, following the main direction of the differential growth, *i.e.* *ψ*_*c*_(*s*, *t*) → *ψ*_*g*_(*s*, *t*). As can be seen from eqs 13 and 14, once an organ is aligned, i.e. *ψ*_*c*_(*s*, *t*) = *ψ*_*g*_(*s*, *t*), there is no movement outside of this plane and only vertical bending is observed. This is also confirmed numerically (shown in S2 Video), where simulations use a an initial curvature and principal direction constant along the organ, *C*(*s*, 0) = *C*_0_ and *ψ*_*c*_(*s*, 0) = *ψ*_0_ (see [29]—key 2). The details of the simulations can be found in the caption of S1 Video.

We now consider a more complex case, where the orientation of the differential growth rotates periodically with a constant angular frequency *ω*. The direction of the differential growth is then given by

$$\psi_{g}\left(s,t\right)=\omega t.$$ (19)

In this case an analytical stability analysis can be performed. The movement of a single element displays a periodic movement, the periodicity of which is given by the direction of the differential growth *ψ*_*g*_(*t*) (see S1 Text section 1.2 and Fig A therein). Furthermore the stability and periodicity are independent of the initial conditions. This means that even when the rotation is not centered around the base of the organ, the pattern remains stable and the periodicity is still given by the internal oscillator (simulations giving rise to a circular pattern are shown in S3 Video).

**Fig 5:**
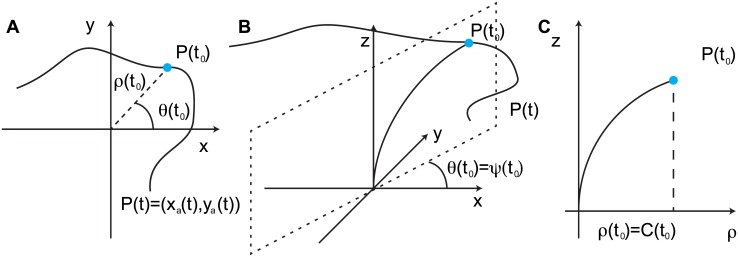
**A. The trajectory of the apical tip projected onto the** (*x*, *y*) **plane produces a parametric curve** *P*(*t*) = (*x*_*a*_(*t*), *y*_*a*_(*t*)). Under the assumptions H1–H3, the position of the apical projection *P*(*t*), gives information on the amplitude and the direction of the plane of curvature, in polar coordinates (*ρ*(*t*), *θ*(*t*_0_)). B. At a given time *t*_0_ the organ is considered to be curved with a constant curvature *C*(*t*_0_) along the organ in a single plane defined by the angle *ψ*(*t*_0_) = *θ*(*t*_0_). The measurement of *θ*(*t*), the angle of the projected apical curve, is then equivalent to *ψ*_*c*_(*t*) C. Inside the plane perpendicular to the horizontal plane that contains the organ, the curvature can be measured directly as the value of *ρ*(*t*_0_) (see S1 Text section 2–4 for a mathematical justification and a description of the approximations).

As mentioned earlier, existing experimental observations concerning nutation measure the movement of the apical tip in the horizontal (*x*, *y*) plane, in the form of a parametric curve *P*(*t*) = (*x*_*a*_(*t*), *y*_*a*_(*t*)). In order to interpret existing data, we analyze the projected movement in the context of our suggested model. Under the hypotheses H1–H3 measurements in the horizontal plane should give direct information on the dynamics of the plant if its shape is known. According to H1, the simplest case is considered, where the dynamics do not depend on the local position along the organ, the curvature is the same along the organ, *C*(*s*, *t*) = *C*(*t*) and the entire organ is curved inside the same plane, *ψ*_*c*_(*s*, *t*) = *ψ*_*c*_(*t*). Therefore there is no dependence on space, and the material derivative $\frac{D}{Dt}$, in Eq 7, is equivalent to the partial derivative $\frac{\partial }{\partial t}$. The projection of the apical tip in the horizontal plane (*x*_*a*_(*t*), *y*_*a*_(*t*)), or (*ρ*(*t*), *θ*(*t*)) in polar coordinates (Fig 5), is then a direct approximation of *ψ*_*c*_(*t*) and *C*(*t*) since by definition *ψ*_*c*_(*t*) = *θ*(*t*) (Fig 5) and *C*(*t*) = *ρ*(*t*) (see S1 Text section 3). A direct estimate of the principal direction of the differential growth *ψ*_*g*_(*t*) can be obtained from the coordinates (*x*_*a*_(*t*), *y*_*a*_(*t*)) of the projection of the apical tip in the horizontal plane (see S1 Text section 4):

$$\psi_{g}\left(t\right)=\arctan\left(\frac{dx_{a}\left(t\right)}{dy_{a}\left(t\right)}\right),$$ (20)

as well as an estimate of the differential growth term (see S1 Text 4):

$$\Delta \left(\psi_{g}\left(t\right),t\right)\dot{E}\left(t\right)=\frac{2R}{L}\sqrt{{\left(\frac{dx_{a}\left(t\right)}{dt}\right)}^{2}+{\left(\frac{dy_{a}\left(t\right)}{dt}\right)}^{2}},$$ (21)

where *L* is the length of the organ. In most of the published data, *L* is not available, meaning that values of $\Delta \left(\psi_{g}\left(t\right),t\right)\dot{E}\left(t\right)$ measured from the horizontal plane can only be compared qualitatively up to a prefactor. On the other hand Eq 20 is independent of *L*, and the principal direction of growth *ψ*_*g*_(*t*) can be measured quantitatively from the observed pattern.

## Results

**Fig 6:**
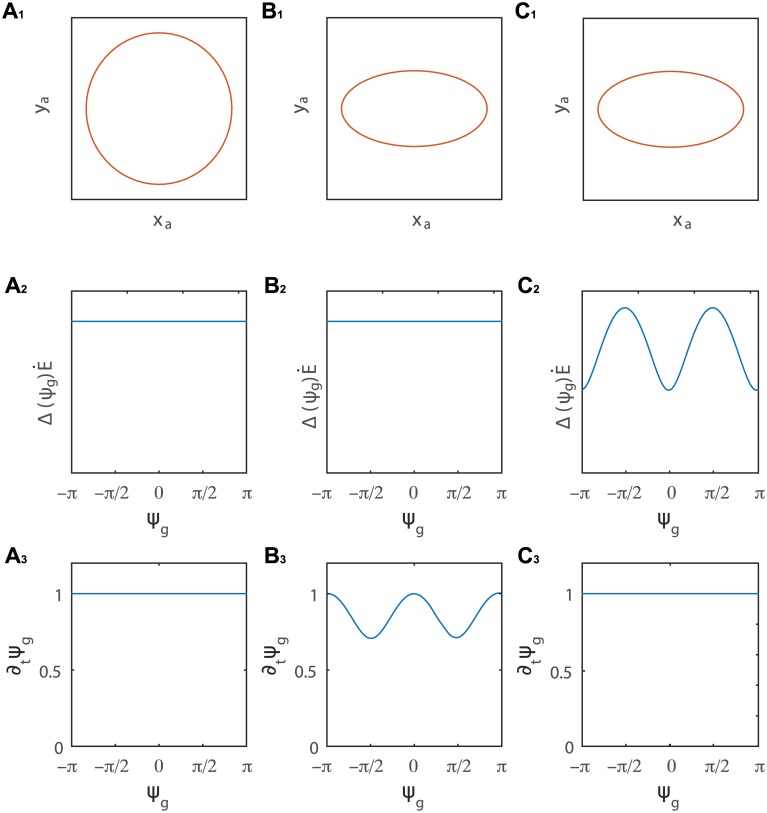
**A. Mechanism underlying a circular pattern**: *A*_1_. A circular pattern in the plane of the apical tip defined by (*x*_*a*_(*t*), *y*_*a*_(*t*)). This is realized by a constant differential growth $\Delta \left(\psi_{g}\left(t\right),t\right)\dot{E}\left(t\right)$ (*A*_2_) and constant variation of the direction of the differential growth $\frac{d\psi_{g}\left(t\right)}{dt}$ (*A*_3_). A simulation of an organ with these conditions can be found in S3 Video. B and C (see [29]—key 0 and 1). Underlying mechanisms for an elliptic pattern: *B*_1_ and *C*_1_. An ellipse in the horizontal plane. This pattern can be realized with a constant constant $\Delta \left(\psi_{g}\left(t\right),t\right)\dot{E}\left(t\right)$ (*B*_2_) and a periodically varying $\frac{d\psi_{g}\left(t\right)}{dt}$ which is maximal close to the major axis of the ellipse (*B*_3_). A simulation can be found in S4 Video. Another option is if $\Delta \dot{E}$ varies periodically and is maximal close to the major axis of the ellipse (*C*_2_), even if $\frac{d\psi_{g}\left(t\right)}{dt}$ is constant in time (*C*_3_). A simulation is presented in S5 Video. A combination of these two limiting mechanisms will also lead to an ellipse.

In what follows we re-examine existing experimental observations in the context of our model. We analyze different classes of movements recorded in the horizontal plane, examining possible underlying mechanisms. We first consider the simulated apical trajectories of the most common observed patterns, the circle and the ellipse. Fig 6 presents the underlying form of the variation of the principal direction of growth $\frac{d\psi_{g}\left(t\right)}{dt}$ and its differential growth $\Delta \left(\psi_{g}\left(t\right),t\right)\dot{E}\left(t\right)$, as imposed by the model *via* eqs 20 and 21. In the case when the apical tip draws a circle in the horizontal plane, it follows that $\frac{d\psi_{g}\left(t\right)}{dt}$ and $\Delta \left(\psi_{g}\left(t\right),t\right)\dot{E}\left(t\right)$ are constant in time (shown in Fig 6A). In the case of an ellipse, there are two possible mechanisms: (i) a periodic $\frac{d\psi_{g}\left(t\right)}{dt}$ with maxima at some $\psi_{g}^{0}$ and $\psi_{g}^{0}+\pi$ (Fig 6B), meaning that the direction of differential growth changes faster on opposite sides of the organ, giving less time for the organ to curve out in those directions, resulting in a smaller radius at those ends. (ii) a periodic $\Delta \left(\psi_{g}\left(t\right),t\right)\dot{E}\left(t\right)$ (Fig 6C) with maxima at some $\psi_{g}^{0}+\pi /2$ and $\psi_{g}^{0}+\pi /2$, meaning that the differential growth is larger at opposite sides of the organ, resulting in a greater curvature and therefore also larger radius at those ends (simulations giving rise to these patterns are given in S3, S4 and S5 Videos).

For the sake of intuition, Fig 6 presents limit cases where either $\frac{d\psi_{g}\left(t\right)}{dt}$ or $\Delta \left(\psi_{g}\left(t\right),t\right)\dot{E}\left(t\right)$ are periodic while the other is constant, however in reality both may be periodic, and their relative magnitude and shift in phase will dictate the final apical pattern. We also note that taking the average value of $\frac{d\psi_{g}\left(t\right)}{dt}$ yields the time it takes for the direction of differential growth to make a full rotation of the organ, i.e.

$$T_{r}=2\pi /\left\langle \frac{d\psi_{g}\left(t\right)}{dt}\right\rangle .$$ (22)

If $\frac{d\psi_{g}\left(t\right)}{dt}$ itself exhibits periodicity, we expect the time between two maxima to coincide with this period, since consecutive maxima are expected to be an angle of *π* apart. Moreover, we note that $\frac{d\psi_{g}\left(t\right)}{dt}$ and $\Delta \left(\psi_{g}\left(t\right),t\right)\dot{E}\left(t\right)$ are plotted here as a function of *ψ*_*g*_, assuming the organ does not exhibit torsion, i.e. the rotation of the cotyledon on top of the organ’s movement, in which case the behavior would be shifted leading to erroneous conclusions. On the other hand a periodic or constant behavior would still be observed when plotting these values as a function of time. Lastly, as the sign of $\Delta \left(\psi_{g}\left(t\right),t\right)\dot{E}\left(t\right)$ does not change, one cannot distinguish between variation of the median elongation, which is supposed to be positive, and variation of the differential growth term.

**Fig 7:**
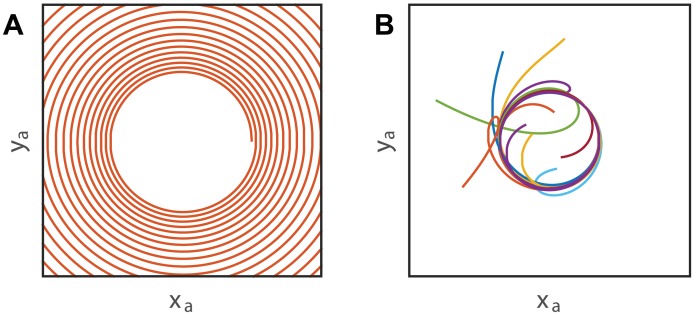
*A*. **The effect of growth on the apical movement in the horizontal plane**. A spiral is observed due to the increase in size of the organ. *B* When proprioception is added to a circular pattern, (see Fig 6.A), the apical tip modifies its trajectory to turn on a circular pattern centered on the base of the organ. Each colors represent different initial conditions.

Let us now consider the effect of elongation on the observed patterns. In the case where the whole organ is growing, *L* < *L*_*gz*_, the curvature does not change as the organ grows, but the increasing length of the organ results in a spiral (Fig 7.A). The time to make a full turn remains unchanged, as Eq 22 is independent of the length of the organ (see [29]—key 4). In the case where *L* > *L*_*gz*_, the pattern remains circular, however the part of the organ outside of the growth zone is fixed in a helical pattern, and the organ is curved in different directions. The observed circular pattern will exhibit a drift. If this helix is small, *CL*_*gz*_ ≪ *R*/*L*_*gz*_, it may not be noticeable experimentally (see [29]—key 5).

No experimental account of this kind of helical pattern has been reported, suggesting a strong regulation of the curvature. Following the case of gravitropism [14], proprioception is a good candidate for curvature regulation. A proprioceptive term can easily be added to Eq 13:

$$\Delta_{\|}\left(s,t\right)=-\gamma C+\Delta \left(\psi_{g}\left(s,t\right)\right)\sin\left(\psi_{g}\left(s,t\right)-\psi_{c}\left(s,t\right)\right)$$ (23)

The results obtained for a circular pattern are then slightly modified (Fig 7.B). Here the curvature of the organ is reduced by proprioception, which tends to straighten the organ [11]. The observed pattern is shifted around the base of the organ, in order to reduce the maximal curvature reached by the organ. The apical tip now converges towards a single stable orbit centered around the base that is fully independent of the initial conditions (see [29]—key 3). Due to the evidence that proprioception prevents fixed curvature in the case of gravitropism [14], it is reasonable to postulate that such regulation is also sufficient in the case of nutation.

**Fig 8:**
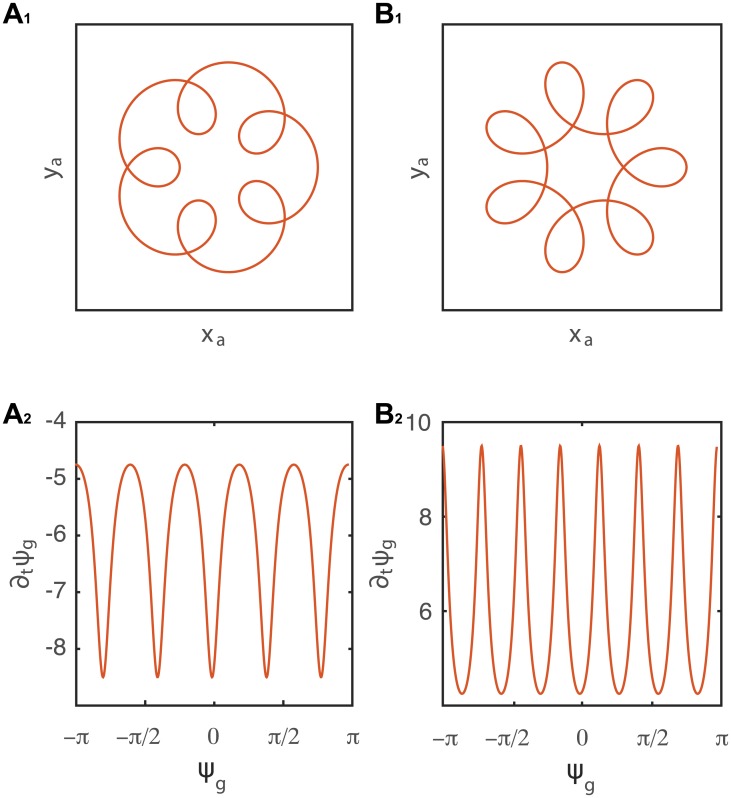
Epi- and hypo-trochoids constructed as a sum of two linear oscillators with angular frequencies respectively *ω*_1_ and *ω*_2_. *A*_1_ *ω*_2_ = 6*ω*_1_ and *B*_1_ *ω*_2_ = −6*ω*_1_. Plotting ∂_*t*_ *ψ*_*g*_ as a function of time is governed by the oscillator with the higher frequency, in this case *ω*_2_ (*A*_2_ and *B*_2_). Simulations of these patterns are presented in S6, S7 and S8 Videos. The analysis of $\frac{d\psi_{g}\left(t\right)}{dt}$ using Eq 20 does not allow to extract accurately the values of *ω*_1_ and *ω*_2_, but rather gives and *effective* oscillator.

Some experimental observations have shown the existence of epi- and hypo-trochoid patterns (spirograph pattern) [22]. These patterns provide an interesting case where the validity of hypothesis H1 (which assumes no local effects) is put in question. Mathematically, a trochoid is constructed as a sum of linear oscillators. If two segments of the organ of length *L*_1_ and *L*_2_ possess different temporal behaviors of the orientation of differential growth *ψ*_*g*1_(*t*) = *ω*_1_*t* and *ψ*_*g*2_(*t*) = *ω*_2_*t*, a trochoid will then be observed in the horizontal plane, as shown in Fig 8, and from the simulations presented in S6, S7 and S8 Videos. Applying Eq 20 to the apical curves cannot discern between the two separate oscillators, and will therefore result in an *effective* *ψ*_*g*_(*t*). Furthermore, the sign of the effective $\frac{d\psi_{g}\left(t\right)}{dt}$ is dominated by the faster oscillator.

**Fig 9:**
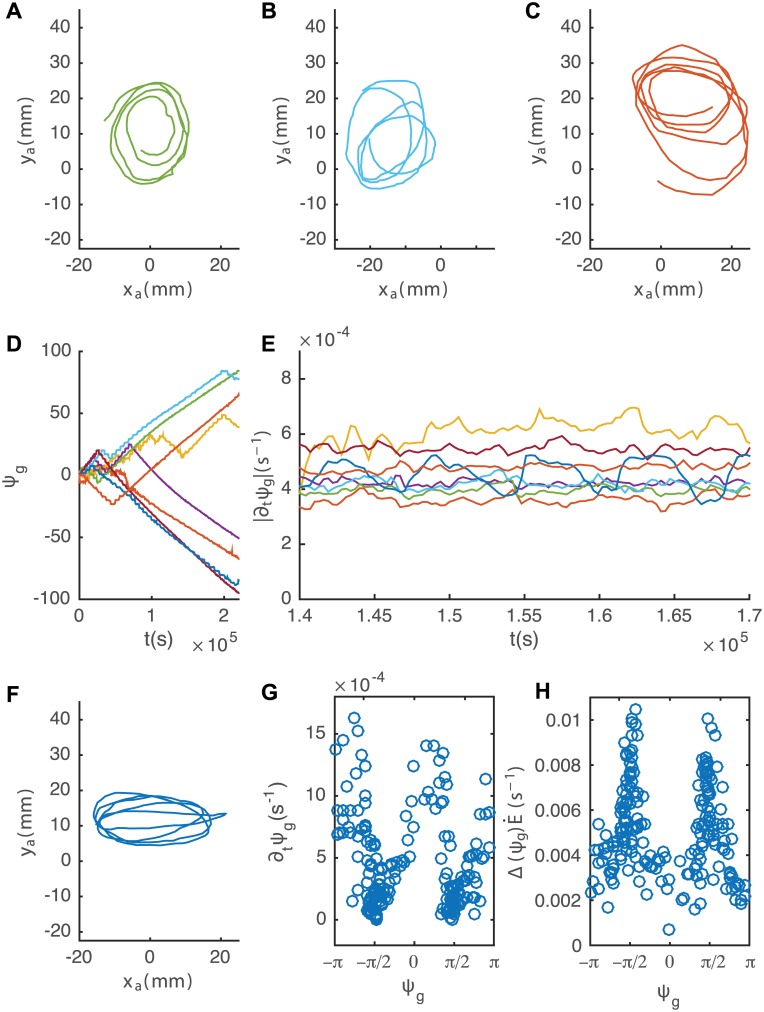
**A. B. C. and F. Four nutation patterns produced by different plants in the horizontal plane during 63 hours, taken from Stolarz et al** [18]. D. The principal direction of differential growth *ψ*_*g*_(*t*) as a function of time. E. Evolution of $\frac{d\psi_{g}\left(t\right)}{dt}$ as a function of *t*. F. An elliptical pattern produced by a plant in the horizontal plane, not exhibiting torsion. G. $\frac{d\psi_{g}\left(t\right)}{dt}$ as a function of *ψ*_*g*_(*t*), showing dips at around *ψ*_*g*_ = ±*π*/2, predicting an elliptical pattern. H.$\Delta \left(\psi_{g}\left(t\right),t\right)\dot{E}\left(t\right)$ as a function of *ψ*_*g*_(*t*), exhibiting peaks at *ψ*_*g*_ = ±*π*/2, in line with the underlying mechanism for an elliptic pattern.

We now analyze an existing dataset of apical movements of 8 *Arabidopsis thaliana* plants published by Stolarz et al. [18] (see Fig 9A, 9B, 9C and 9F for examples of measured apical patterns. Most of the observed patterns are elliptical. We apply eqs 20 and 21 on all 8 measurements of the apical tip in the horizontal plane (*x*_*a*_, *y*_*a*_), extracting *ψ*_*g*_(*t*) (shown in Fig 9D) and $\Delta \left(\psi_{g}\left(t\right),t\right)\dot{E}\left(t\right)$. After ∼20 hours most plants exhibit a linear behavior, equivalent to a constant time derivative $\frac{d\psi_{g}\left(t\right)}{dt}$. Averaging over time and over the different plants results in $\langle \frac{d\psi_{g}\left(t\right)}{dt}\rangle =4.6\times 10^{-4}\pm 7\times 10^{-5}s^{-1}$, and substituting this in Eq 22 gives the time taken for a full rotation of the differential growth direction, *T*_*r*_ = 230 ± 40 min. At this point we focus on a single curve (plotted in dark blue), where no torsion has been observed. Examining $\frac{d\psi_{g}}{dt}$ for closely (plotted in Fig 9E) we identify oscillations. As mentioned earlier, the model predicts the maxima to be *T*_*r*_/2 apart, representing opposite points along the apical curve. Indeed we find that the average time between every other maximum is *T*_*o*_ = 215 ± 25 min, in agreement with *T*_*r*_ found from the average value of $\frac{d\psi_{g}}{dt}$. Moreover, since this plant does not exhibit torsion, one can plot $\frac{d\psi_{g}\left(t\right)}{dt}$ and $\Delta \left(\psi_{g}\left(t\right),t\right)\dot{E}\left(t\right)$ as a function of *ψ*_*g*_(*t*) (shown in Fig 9G and 9H). As predicted from the model, we find the minima of $\frac{d\psi_{g}\left(t\right)}{dt}$ and the maxima of $\Delta \left(\psi_{g}\left(t\right),t\right)\dot{E}\left(t\right)$ situated at *ψ*_*g*_ = ±*π*/2, representing the farthest sides of the ellipse (on the right and left).

## Discussion

A detailed analysis has now been carried out of the kinematics of differential growth outside of the plane of curvature, and its implications on plant movement. This shows how a classical measurement, here the position of the tip in the horizontal plane, is insufficient to provide a clear picture of the relation between observed movements and the underlying growth mechanisms.

Furthermore this study shows that the kinematics of the out of plane curvature can be described as a simple extension of the kinematics relating curvature in the plane and differential growth [14]. By projecting the differential growth on the planes parallel and perpendicular to the plane of curvature, only one supplementary equation is necessary to describe the full kinematics. This equation relates the orientation of the curvature and the growth in the perpendicular plane. The amplitude of the curvature is modified by the difference in growth rate between the two sides of the organ in the plane of curvature. The orientation of curvature in space is then modified by the differential growth in the perpendicular plane. Only three parameters are necessary to account for the full movement: i. the elongation rate along the median line $\dot{E}\left(s,t\right)$ sets the time scale of the movement, ii. the principal direction of differential growth *ψ*_*g*_(*s*, *t*), the direction in which the differential growth is maximal and iii. the distribution of the differential growth in this direction Δ(*ψ*_*g*_(*s*, *t*))(*s*, *t*). The description of this geometrical framework has been neglected to date, and is a central step to unravel the relation between differential growth and nutation.

In both the case of gravitropism [14] and nutation, the destabilizing effects of growth on the movement are regulated by proprioception. The autonomous capacity of plants to control and regulate their own shape is reinforced as a central element of postural control. During gravitropic movements, it has been shown experimentally that effects due to growth could be neglected due to the strong influence of proprioception [14]. It is then expected that this regulation is sufficient to avoid the effects due to growth during nutation.

The position of the apical tip in the the horizontal plane, perpendicular to gravity, has been central to the study of kinematics. The relevance of this measure has never been clearly discussed and the underlying hypotheses have remained hidden. In particular the relation between the movement of the apical tip and the dynamics of the differential growth, the motor of the movement, is difficult to extract because the full shape of the plants remains unknown. A simple set of hypotheses needs to be properly stated to constrain the relation between shapes and movements. The whole organ is now considered as a block that undergoes the same variation all along the organ. Despite the simplification of the problem, this has proved useful to unravel the underlying dynamics of the differential growth, retaining the general observed behavior. As plants tend to align their curvature orientation *ψ*_*c*_ with the principal direction of growth *ψ*_*g*_, the pattern observed in the horizontal plane can remain a marker of growth. Common observed patterns, like the circle or the ellipse, are then directly related to different oscillating patterns of differential growth. Furthermore, simple input such as an oscillation of the principal direction of growth, can produce robust, stable stereotypical patterns independently of the initial conditions.

Minimal regulation of the movement is necessary to achieve commonly observed patterns like the circle or the ellipse. If measurements in the horizontal plane are useful to understand the kinematics of nutation, they are limited in their scope of analysis. Future studies allowing the measurements of proper 3D kinematics should provide a better understanding of the dynamics of differential growth, and give the exact validity of the measurements performed in the horizontal plane.

In conclusion, we have presented a mathematical description of the kinematics of plant nutation based on the interplay between geometry and differential growth. This framework allows a full 3D analysis of complex observed kinematics, shedding light on the underling mechanism, while revisiting the interpretation of common horizontal measurements of the plant tip.

## Supporting Information

S1 VideoDefinitions.The movie shows the basic definitions shown in Figs 2 and 3, and used in the simulations brought here. The abscissa of a plant organ is defined as starting the base (blue dot at the bottom), and ending at the apex (red dot at the top). When looking at a circular cross-section along the organ, a point on its circumference is defined by an angle *ψ* chosen from an arbitrary starting point. We then define *ψ*_*c*_ as the orientation of the vector normal in the principal direction of curvature **c**(shown here as a green vector at the apex), and *ψ*_*g*_ as the direction of the principal differential growth vector **g**, here shown as a black vector at the apex. Simulations of the nutation movements are carried out by solving eqs 13 and 14, together with Eqs 15 and 16, using the appropriate functions of *ψ*_*g*_(*t*) and $\Delta (\psi_{g}\left(s,t\right))\dot{E}\left(s,t\right)$. An example of a simple circular nutation motion in 3D is presented from various points of view, showing how **c** and **g** are oriented throughout.(MP4)

S2 VideoConstant.$\Delta (\psi_{g}\left(s,t\right))\dot{E}\left(s,t\right)=1$ and *ψ*_*g*_ = 0. The principal direction of curvature aligns with the principal direction of growth. Two different initial conditions of the curvature, *C*(0), are presented to show that the dynamics does not depend on this initial conditions.(MP4)

S3 VideoCircle.$\Delta (\psi_{g}\left(s,t\right))\dot{E}\left(s,t\right)=1$ and *ψ*_*g*_ = *ω*_0_*t*. When the principal direction of growth is revolving around the surface of the organ, the pattern displayed is a circle. Two different initial conditions of the curvature, *C*(0), are presented to show that the dynamics does not depend on this initial conditions.(MP4)

S4 VideoEllipse 2.$\Delta (\psi_{g}\left(s,t\right))\dot{E}\left(s,t\right)=1$ and $\frac{d\psi_{g}}{dt}\;=\;\omega_{0}(1\;+\;.5\text{cos}(2\psi_{g}))$. Here the principal direction of growth is oscillating around the surface of the organ, however this revolution is slower when *ψ*_*g*_ = ±*π*/2, the pattern displayed is an ellipse.(MP4)

S5 VideoEllipse 2.$\Delta (\psi_{g}\left(s,t\right))\dot{E}\left(s,t\right)=\left(1+.5\cos\left(2\psi_{g}\right)\right)$ and *ψ*_*g*_ = *ω*_0_*t*. Here the principal direction of growth is revolving around the surface of the organ, while the intensity of the differential growth element $\Delta (\psi_{g}\left(s,t\right))\dot{E}\left(s,t\right)$ is slower when *ψ*_*g*_ = ±*π*/2. The pattern displayed is still an ellipse but it is rotated by *π*/2 from the pattern obtained in S4 Video.(MP4)

S6 Video Trochoids, definitions. An example of a more complex dynamics that can be obtained when different part of stem are constrained by different dynamics. The organ is here divided into two sections, from the base (in blue) to the purple point (Part 1) and from the purple point to the apical tip (in yellow). The red dot corresponds to the movement observed in the horizontal plane when only the apical tip is tracked. The yellow and purple dots shows the underlying dynamics of, respectively, the Part 1 and 2 of the plants.
(MP4)

S7 VideoEpitrochoids.The definition are the same than in S6 Video. Part 1: $\Delta (\psi_{g}\left(s,t\right))\dot{E}\left(s,t\right)=1$ and *ψ*_*g*_ = *ω*_0_. Part 2: $\Delta (\psi_{g}\left(s,t\right))\dot{E}\left(s,t\right)=5$ and *ψ*_*g*_ = 6*ω*_0_*t*.(MP4)

S8 VideoHypotrochoids.The definition are the same than in S6 Video. But here Part 1: $\Delta (\psi_{g}\left(s,t\right))\dot{E}\left(s,t\right)=1$ and *ψ*_*g*_ = *ω*_0_. Part 2: $\Delta (\psi_{g}\left(s,t\right))\dot{E}\left(s,t\right)=5$ and *ψ*_*g*_ = −6*ω*_0_*t*.(MP4)

S1 Text Appendix. Analytical detail of the calculation performed in this manuscript.
(PDF)
