# Supplementary material for: The Kinematics of Plant Nutation Reveals a Simple Relation between Curvature and the Orientation of Differential Growth
Source: PLoS Comput Biol. 2016 Dec 6;12(12):e1005238. doi: 10.1371/journal.pcbi.1005238 (PMC5140061; doi:10.1371/journal.pcbi.1005238)
Supplement: S1 Text — Analytical detail of the calculation performed in this manuscript. (PDF) [file pcbi.1005238.s009.pdf]

The Dynamics of Plant Nutation Reveals A Simple Relation Between Curvature and the Orientation of Differential Growth - Appendix

Renaud Bastien[1,*], Yasmine Meroz[2]

1 Department of Collective Behaviour, Max Planck Institute for Ornithology and Department of Biology, University of Konstanz, Konstanz, Germany
2 School of Engineering and Applied Sciences, Harvard University, Cambridge, MA 02138, USA.

*** rbastien@orn.mpg.de**

The following dimensionless variables are used:

$$\begin{cases} \dot{E}(s,t)t \to t' \\ C(s,t)R \to C'(s,t), \end{cases} \tag{24}$$

In order to simplify the writing of the equation, the prime indices are dropped. A more compact form to write derivative is also used

$$\begin{cases} \frac{dx}{dy} \to d_y x \\ \frac{\partial x}{\partial y} \to \partial_y x \\ \frac{Dx}{Dy} \to D_y x \end{cases} \tag{25}$$

1 Analysis of the movements induced by the principal direction of growth

Substituting equations 15 and 16 in equations 13 and 14, yields the set of equations:

$$\partial_t C(s,t) = \cos(\psi_g(s,t) - \psi_c(s,t)), \tag{26}$$

$$\partial_t \psi_c(s,t) = \frac{\sin(\psi_g(s,t) - \psi_c(s,t))}{C(s,t)}. \tag{27}$$

Following H1, the dynamics does not depend on the local position along the organ, $C(s,t) \to C(t)$ and $\psi_c(s,t) \to \psi_c(t)$. In following, in order to simplify the writing of the equation, the dependence in time is dropped. The following set of initial conditions is considered:

$$\psi_c(0) = \psi_c^0 \tag{28}$$

$$C(0) = C_0. \tag{29}$$

This accounts for an organ curved in a single plane. The following change of variables is considered

$$u = \psi_c - \psi_g, \tag{30}$$

and substituting in equations 26 and 27 yields:

$$\partial_t C = \cos(u) \tag{31}$$

$$\partial_t u - \partial_t \psi_g = -\frac{\sin(u)}{C}. \tag{32}$$

Rearranging equation 32 gives and expression for C:

$$C = -\frac{\sin(u)}{\partial_t u - \partial_t \psi_g}, \tag{33}$$

which we substitute in equation 31, yielding

$$-\frac{\partial_t u}{\partial_t u - \partial \psi_g} \cos u + \frac{\partial_t^2 u - \partial_t^2 \psi_g}{(\partial_t u - \partial_t \psi_g)^2} \sin u = \cos u, \tag{34}$$

or alternatively after rearranging:

$$(-2\partial_t u + \partial_t \psi_g) \cot u + \frac{\partial_t^2 u - \partial_t^2 \psi_g}{\partial_t u - \partial_t \psi_g} = 0. \tag{35}$$

1.1 Constant principal direction of growth, $\psi_g = 0$

In the simplest case, where the principal direction of growth is constant, and arbitrarily chosen to take the value 0:

$$\psi_g = 0, \tag{36}$$

resulting in $u = \psi_c$, and equation 35 can be rewritten as

$$-2\partial_t \psi_c \cot \psi_c + \frac{\partial_t^2 \psi_c}{\partial_t \psi_c} = 0. \tag{37}$$

Integrating over time yields:

$$-2 \log(\sin \psi_c) + \log(\partial_t \psi_c) + \tilde{K_1} = 0, \tag{38}$$

where $\tilde{K_1}$ is an integration constant. Taking an exponential yields:

$$\partial_t \psi_c = K_1 \sin^2 \psi_c, \tag{39}$$

where $K_1 = e^{-\tilde{K_1}}$. An additional integration over time results in:

$$\cot \psi_c = K_1 t + K_2, \tag{40}$$

where K_2 is another integration constant. Rearranging results in:

$$\psi_c = \arctan\left(\frac{1}{K_1 t + K_2}\right). \tag{41}$$

Substituting the initial condition in equation 28

$$\psi_c(t=0) = \psi_c^0 = \arctan\left(\frac{1}{K_2}\right), \tag{42}$$

yields the value of K_2:

$$K_2 = \cot \psi_c^0. \tag{43}$$

We proceed to extract K_1, substituting equation 39 in equation 27:

$$K_1 \sin^2 \psi_c = -\frac{\sin \psi_c}{C}. \tag{44}$$

Substituting the initial conditions stated in equations 28 and 29 yields:

$$K_1 \sin^2 \psi_c^0 = -\frac{\sin \psi_c^0}{C_0}, \tag{45}$$

$$K_1 = -\frac{1}{C_0 \sin \psi_c^0}. \tag{46}$$

We now substitute the expressions for K_1 and K_2 (found in equations 46 and 43) in equation 41, leading to the final expression for $\psi_c(t)$:

$$\psi_c = \arctan\left(\frac{1}{\cot \psi_c^0 - t/(C_0 \sin \psi_c^0)}\right). \tag{47}$$

Note that for long times, $t \to \infty$, the direction of maximal curvature is equal to the principal direction of growth, $\psi_c = \psi_g = 0$.

1.2 Temporal linear variation of the principal direction of growth, $\psi_g = \omega t$

We continue with the linear case

$$\psi_g = \omega t. \tag{48}$$

Equation 35 now takes the form:

$$(-2\partial_t u + \omega) \cot u + \frac{\partial_t^2 u}{\partial_t u - \omega} = 0. \tag{49}$$

Integrating over time yields (with K' is an integration constant):

$$-2 \log \sin u + \log\left(\partial_t u - \omega\right) = K' - \omega \int^t \cot u dt', \tag{50}$$

and raising to an exponent and rearranging yields:

$$\partial_t u = K e^{-\omega \int^t \cot u dt'} \sin^2 u + \omega, \tag{51}$$

where $K = e^{K'}$. We substitute equation 51 in equation 27, and use the initial conditions in equations 28 and 29, leading to:

$$K \sin^2 \psi_c^0 = -\frac{\sin \psi_c^0}{C_0}, \tag{52}$$

and rearranging this yields the integration constant K:

$$K = -\frac{1}{C_0 \sin \psi_c^0}. \tag{53}$$

In order to solve the non linear equation 51, the following change of variable is considered:

$$v = \cot u, \tag{54}$$

with

$$dv = -\frac{du}{sin^2 u}. \tag{55}$$

With this change of variables equation 51 now takes the form:

$$\partial_t v = -K_1 \, e^{-\omega \int^t v dt'} - \omega\left(1 + v^2\right), \tag{56}$$

and taking the logarithm yields:

$$\log\left(\partial_t v + \omega\left(1 + v^2\right)\right) = \omega \int^t v dt' + K_1. \tag{57}$$

Taking the derivative of time yields:

$$\frac{\partial_t^2 v + 2\omega v \partial_t v}{\partial_t v + \omega\left(1 + v^2\right)} = \omega v, \tag{58}$$

and after rearranging:

$$\partial_t^2 v + \omega v \partial_t v - \omega^2 v(1 + v^2) = 0. \tag{59}$$

We now introduce a last change of variables:

$$\tau = \omega t, \tag{60}$$

leading to the following form:

$$\partial_\tau^2 v + v \partial_\tau v - v(1 + v^2) = 0. \tag{61}$$

This equation is then the solution, where we remind that $v = \cot\left(\psi_c - \psi_g\right)$. The phase field of this equation is given for different values of the initial condition. Stable orbits are observed in Figure S1. The movement of a single element displays a stable orbit, the periodicity of which is given by the direction of the differential growth ω. Furthermore the stability and periodicity are independent of the initial conditions. This means that even when the rotation is not centered around the base of the organ, the pattern remains stable and the periodicity is still given by the internal oscillator.

2 Measuring the curvature C from the base-apex distance in the apical plane ρ_a

Existing experimental measurements are usually restricted to the apical plane, therefore disregarding important information concerning the conformation of the whole shoot, needed for the analysis presented here. In what follows we show that one can recover the curvature of the shoot C (assumed here to be constant along the shoot) from ρ_a, the distance of the apex point from the base in the apical plane, as depicted in Fig.3.C. This can be done through a purely geometric argument, whose details appear in Figure S2.

Assuming a constant curvature C along the shoot, we can consider the shoot an arc, part of a circle. The radius of this circle is the inverse of the curvature by definition $R = 1/C$, and the length of the arc is just L the length of the shoot. We can therefore calculate the angle defined by the arc, γ.

$$\gamma = LC. \tag{62}$$

Drawing a straight line between the base and apex, we now have an isosceles triangle with equal base angles α and the third angle positioned at the center of the circle of size γ. Therefore the sum of angles yields:

$$2\alpha + \gamma = \pi/2 \tag{63}$$

The angle between this base-apex line and the projection of the apex to the ρ_a axis is denoted $beta$, defining a right angle triangle, i.e.:

$$\alpha + \beta = \pi/2. \tag{64}$$

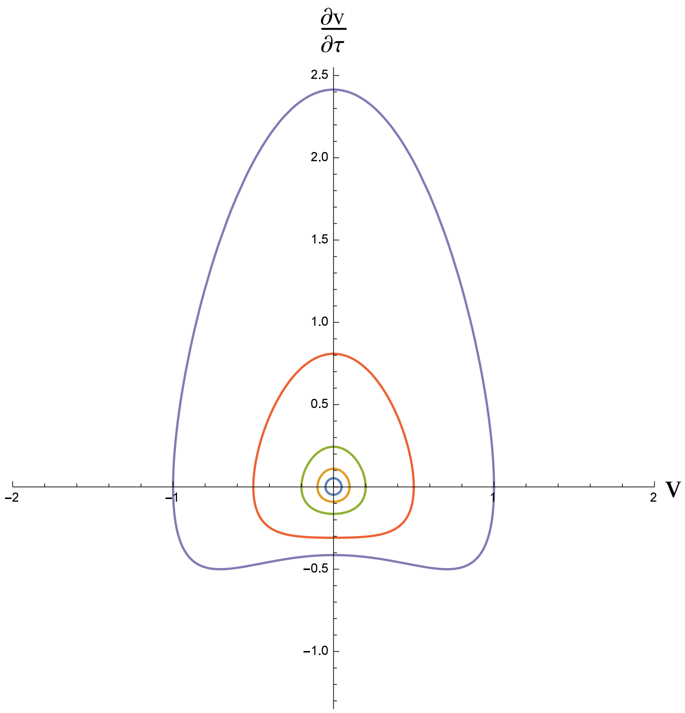

Figure S1. The solutions of equation 61 are represented in the space $(v, \partial_\tau v)$, for the following initial conditions blue $v = 0.05$, yellow $v = 0.1$, green $v = 0.2$, red $v = 0.5$, dark blue $v = 1$. Orbits are observed and are all travelled during a time $\tau = 2\pi$. This means that after a time $\tau = 2\pi$, the system comes back to the same initial condition. The orbits are then stable are the same pattern will be displayed at longer times. As v becomes bigger, it i.e. the phase between ψ_g and ψ_c is different from $\pi/2$ the dynamics is not symmetrical anymore. However the pattern observed in the apical plane remain the same (*e.g.* compared MovieS2 and S3)

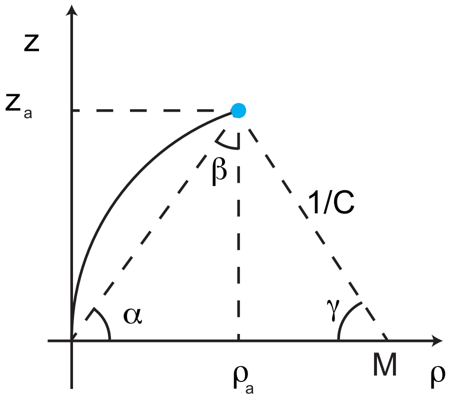

Figure S2. Definition of the variables used to measure the curvature in the apical plane. The organ is considered to have a constant curvature C so that the point M on the rho abscissa is the center of the circle described by the organ. The distance from M to the apical tip, (ρ_a, z_a) in the plane (ρ, z), or to the basis of the organ,(0,0), is the radius of curvature $1/C$.

Substituting equation 64 in equation 63 results in;

$$\gamma = 2\beta. \tag{65}$$

By definition,

$$\tan\beta = \rho_a/z_a, \tag{66}$$

and substituting equations 65 and 62 yields:

$$\tan\frac{CL}{2} = \frac{\rho_a}{z_a}. \tag{67}$$

For small curvatures $CL \ll 1$, we can approximate $\tan(CL/2) \sim CL/2$, and $z_a \sim L$, leading to

$$C = 2L^{-2}\rho_a, \tag{68}$$

3 Measuring ψ_c in the apical plane and its relation to ψ_g

The orientation of the principal direction of curvature of an organ is defined in the apical plane

$$\psi_c = \arctan\left(\frac{x_a}{y_a}\right). \tag{69}$$

The differential element $d\psi_c$ is then given by

$$d\psi_c = \frac{d(x_a/y_a)}{1 + x_a^2/y_a^2}, \tag{70}$$

or after rearranging:

$$d\psi_c = \frac{dy_a}{y_a}\frac{dx_a/dy_a - x_a/y_a}{1 + x_a^2/y^2}. \tag{71}$$

The distance from the apex to the base in the apical plane, ρ_a, is defined by the position of the apex in that plane, (x_a, y_a), and substituting equation 68 results in:

$$\frac{L^4}{4}C^2 = \rho_a^2 = x_a^2 + y_a^2. \tag{72}$$

The differential is then:

$$\frac{L^4}{4}CdC = x_a dx_a + y_a dy_a. \tag{73}$$

Dividing this by equation 72 and rearranging yields:

$$\frac{dC}{C} = \frac{dy_a}{y_a}\frac{(dx_a/dy_a)(x_a/y_a) + 1}{1 + x_a^2/y_a^2}, \tag{74}$$

where we note that the prefactor $\frac{L^4}{4}$ has canceled out. Dividing equation 27 by equation 26 results in:

$$\psi_g - \psi_c = \arctan\left(\frac{C\partial_t\psi_c}{\partial_t C}\right), \tag{75}$$

and substituting equations 71 and 74 yields:

$$\psi_g - \psi_c = \arctan\left(\frac{dx_a/dy_a - x_a/y_a}{(dx_a/dy_a)(x_a/y_a) + 1}\right). \tag{76}$$

We now use the identity $\arctan\left(\frac{u-v}{uv+1}\right) = \arctan(u) - \arctan(v)$, and substituting equation 69, yields:

$$\psi_g = \arctan\left(\frac{dx_a}{dy_a}\right). \tag{77}$$

This can be expressed in terms of the temporal derivative of x_a and y_a, finally yielding equation 14 in the main text:

$$\psi_g(t) = \arctan\left(\frac{d_t x_a(t)}{d_t y_a(t)}\right). \tag{78}$$

$$\psi_g(t) = \arctan\left(\frac{dx_a(t)}{dy_a(t)}\right). \tag{79}$$

4 Measuring $\Delta(\psi_g)\dot{E}$ in the apical plane

Substituting equations 71, 72 and 73 into equation 12, we obtain the following relation

$$\Delta(\psi_g(s,t))\dot{E}(s,t) = \sqrt{4L^{-4}\frac{(x_a\partial_t x_a + y_a\partial_t y_a)^2}{x_a^2+y_a^2}R^2 + 4L^{-4}(x_a^2+y_a^2)\left(\frac{y_a\partial_t x_a - x_a\partial_t y_a}{y_a^2+x_a^2}\right)^2 R^2}, \tag{80}$$

which yields

$$\Delta(\psi_g(s,t))\dot{E}(s,t) = 2L^{-2}R\sqrt{\frac{(x_a\partial_t x_a + y_a\partial_t y_a)^2 + (y_a\partial_t x_a - x_a\partial_t y_a)^2}{x_a^2+y_a^2}}. \tag{81}$$

and after rearranging

$$\Delta(\psi_g(s,t))\dot{E}(s,t) = 2L^{-2}R\sqrt{\partial_t x_a(t)^2 + \partial_t y_a(t)^2}. \tag{82}$$
